# Toward a common standard for data and specimen provenance in life sciences

**DOI:** 10.1002/lrh2.10365

**Published:** 2023-04-18

**Authors:** Rudolf Wittner, Petr Holub, Cecilia Mascia, Francesca Frexia, Heimo Müller, Markus Plass, Clare Allocca, Fay Betsou, Tony Burdett, Ibon Cancio, Adriane Chapman, Martin Chapman, Mélanie Courtot, Vasa Curcin, Johann Eder, Mark Elliot, Katrina Exter, Carole Goble, Martin Golebiewski, Bron Kisler, Andreas Kremer, Simone Leo, Sheng Lin‐Gibson, Anna Marsano, Marco Mattavelli, Josh Moore, Hiroki Nakae, Isabelle Perseil, Ayat Salman, James Sluka, Stian Soiland‐Reyes, Caterina Strambio‐De‐Castillia, Michael Sussman, Jason R. Swedlow, Kurt Zatloukal, Jörg Geiger

**Affiliations:** ^1^ BBMRI‐ERIC Graz Austria; ^2^ Institute of Computer Science & Faculty of Informatics Masaryk University Brno Czechia; ^3^ CRS4—Center for Advanced Studies Research and Development in Sardinia Pula Italy; ^4^ Medical University Graz Graz Austria; ^5^ National Institute of Standards and Technology Gaithersburg Maryland USA; ^6^ Biological Resource Center of Institut Pasteur (CRBIP) Paris France; ^7^ EMBL's European Bioinformatics Institute (EMBL‐EBI) Cambridge UK; ^8^ Plentzia Marine Station (PiE‐UPV/EHU) University of the Basque Country, EMBRC‐Spain Bilbao Spain; ^9^ University of Southampton Southampton UK; ^10^ King's College London London UK; ^11^ Ontario Institute for Cancer Research Toronto Ontario Canada; ^12^ University of Klagenfurt Klagenfurt Austria; ^13^ Department of Social Statistics, School of Social Sciences University of Manchester Manchester UK; ^14^ Flanders Marine Institute (VLIZ), EMBRC‐Belgium Ostend Belgium; ^15^ Department of Computer Science University of Manchester Manchester UK; ^16^ Heidelberg Institute for Theoretical Studies (HITS gGmbH) Heidelberg Germany; ^17^ Independent consultant; ^18^ ITTM S.A. Esch‐sur‐Alzette Luxembourg; ^19^ Biosystems and Biomaterials Division NIST Gaithersburg Maryland USA; ^20^ Department of Biomedicine University of Basel Basel Switzerland; ^21^ SCI‐STI‐MM École Politechnique Fédérale de Lausanne Lausanne Switzerland; ^22^ Centre for Gene Regulation and Expression and Division of Computational Biology, School of Life Sciences University of Dundee Dundee UK; ^23^ German BioImaging–Gesellschaft für Mikroskopie und Bildanalyse e.V. Konstanz Germany; ^24^ Japan bio‐Measurement and Analysis Consortium Tokyo Japan; ^25^ INSERM–Institut National de la Sante et de la Recherche Medicale Paris France; ^26^ Standards Council of Canada Ottawa Ontario Canada; ^27^ Canadian Primary Care Sentinel Surveillance Network (CPCSSN) Department of Family Medicine Queen's University Kingston Ontario Canada; ^28^ Biocomplexity Institute Indiana University Bloomington Indiana USA; ^29^ Informatics Institute University of Amsterdam Amsterdam The Netherlands; ^30^ Program in Molecular Medicine University of Massachusetts Chan Medical School Worcester Massachusetts USA; ^31^ US Department of Agriculture Washington District of Columbia USA; ^32^ Interdisciplinary Bank of Biomaterials and Data Würzburg (ibdw) Würzburg Germany

**Keywords:** biotechnology, International Organization for Standardization, provenance information, standardization

## Abstract

Open and practical exchange, dissemination, and reuse of specimens and data have become a fundamental requirement for life sciences research. The quality of the data obtained and thus the findings and knowledge derived is thus significantly influenced by the quality of the samples, the experimental methods, and the data analysis. Therefore, a comprehensive and precise documentation of the pre‐analytical conditions, the analytical procedures, and the data processing are essential to be able to assess the validity of the research results. With the increasing importance of the exchange, reuse, and sharing of data and samples, procedures are required that enable cross‐organizational documentation, traceability, and non‐repudiation. At present, this information on the provenance of samples and data is mostly either sparse, incomplete, or incoherent. Since there is no uniform framework, this information is usually only provided within the organization and not interoperably. At the same time, the collection and sharing of biological and environmental specimens increasingly require definition and documentation of benefit sharing and compliance to regulatory requirements rather than consideration of pure scientific needs. In this publication, we present an ongoing standardization effort to provide trustworthy machine‐actionable documentation of the data lineage and specimens. We would like to invite experts from the biotechnology and biomedical fields to further contribute to the standard.

## 1 INTRODUCTION

The profound crisis of scientific reproducibility has its roots in the enhanced availability of large volumes of data that are produced at ever‐increasing velocity, which in turn often leads to the dissolution of the control mechanisms that traditionally ensured the quality of data and processes.^1^, ^2^, ^3^, ^4^, ^5^, ^6^, ^7^, ^8^, ^9^, ^10^, ^11^ At the same time, the origin and history of specimens used to generate research data often remain inexplicit. While considerable effort has been put into the development of standards for specimen quality, the actual documentation has been left to the discretion of the provider of the specimen and data. As a result, the situation is exacerbated by the lack of consistent and comprehensive documentation of specimens and data, which could support the identification of suspected, or proven use of, fabricated data or specimens of unclear origin. Hence, the urgent need for trustworthy documentation of the data lineage and specimens is evident, especially when considering the serious impact of irreproducible or even flawed scientific results on health, economics, and political decisions.^12^, ^13^, ^14^, ^15^, ^16^

It is generally accepted that the reliability of data generated in downstream analytical procedures^17^, ^18^, ^19^ is significantly impacted by the properties and quality attributes of specimens, which are precursors of the data. Experts from multiple life sciences domains have called for the improvement and standardization of the documentation of research and scientific service processes.^20^, ^21^, ^22^, ^23^, ^24^, ^25^, ^26^ This has led in turn to the progressive development and implementation of data management and other functional tools, such as discovery services, access pipelines, and standardized data models, enabling the sharing of data and specimens.^27^, ^28^, ^29^, ^30^, ^31^, ^32^ In practice, however, there remains a gap between the needs and the reality of the requirements specified in accepted standards, including technical, operational, and legal specifications needed to ensure the trustworthiness and traceability of data and specimens. In an effort to remedy these deficiencies in the provenance captured and reported, we are endeavoring to develop an *international standard on provenance information system for the life sciences* accepted by both academia and industry. Provenance information can be used to assess the quality and reliability, and hence the reusability of the object, that is, the data, the metadata, the biological materials, or the specimens.

### 1.1 Objectives for a provenance standard

One of the main characteristics of present‐day research in life sciences is that the research objects, such as datasets or specimens, are exchanged between organizations. Therefore, each of the organizations involved can only provide documentation for a part of the object's life cycle. Consequently, an uninterrupted chain of provenance information documenting the whole life cycle can only be formed from individual parts of provenance distributed across different sources. To enable meaningful integration and harmonized processing of the distributed provenance parts, semantic interoperability between standalone distributed provenance parts must be ensured. In addition, the processing of the resulting chain of distributed provenance must be designed to (a) deal with missing provenance components in the chain, so the chain is not interrupted or corrupted when an intermediary organization has not generated appropriate provenance information, or if the organization ceased to exist; (b) handle sensitive or confidential information contained in provenance information, keeping it opaque and disclosed only by authorization; (c) handle several versions of the same provenance information, for instance, when an error in provenance is found and is fixed; and (d) enable verification of the integrity and authenticity of provenance components, even for opaque provenance components, to ensure the trustworthiness of provenance.

The distributed provenance chain must be suited to answer essential queries independent of the research domain, such as *“What are the precursors of a given dataset?”* or *“Which processes precede a given dataset creation?”*. The underlying query resolution mechanism must be able to navigate through the chain, regardless of the actual site where the corresponding part of the distributed provenance is stored, which processes or objects are documented, or what the actual source of the provenance is.

The provenance standard must therefore include a general concept, providing a basis for common aspects shared between various domains which are part of the life cycle of a documented research object. In particular, these common aspects include (a) traversing distributed provenance chains; (b) implementing domain‐independent properties for the provenance, such as confidentiality, authenticity, integrity, non‐repudiation, and validity; and (c) locating a specific part of provenance in the distributed provenance. In addition, support for any domain‐specific aspect, such as quality‐related queries, must be provided and aligned with the common foundation without disrupting the general properties of the chain.

## 2 RESULTS AND DISCUSSION

The novelty of the proposed standard is that it is the first provenance information standard for the biomedical domain that aims to address the aforementioned requirements. In addition, the standard covers both, physical and digital objects and links them to a common provenance chain, while ensuring the common properties of resulting provenance parts. It supports fully distributed provenance information management and aims to handle a wide range of complex real‐world scenarios. As part of the standard development, we have proposed the Common Provenance Model (CPM),^33^ which forms the conceptual foundation of the standard. The CPM is the only provenance model that provides a baseline for distributed provenance chains, as they were described above.

The need for an effort to address the issues in provenance was proposed to the International Standards Organization (ISO) Technical Committee 276 “Biotechnology” (ISO/TC 276) in 2017 and approved as a preliminary work item. In 2020, ISO/TC 276 approved a new work item proposal to develop an international standard for biological material and data provenance which is registered as a committee draft, ISO/DTS 23494‐1 *Biotechnology*—*Provenance information model for biological material and data*—*Part 1: Design concepts and general requirements*. To the best of our knowledge, this standard is the first provenance information standard for the biotechnology domain, addressing the need for consistent documentation of the life‐cycle of related research objects from the acquisition of a specimen to analytical procedures and downstream data processing and analysis. This standard is conceptualized according to the FAIR principles,^34^ which provide high‐level methodological recommendations, including guidance on provenance.* As the FAIR principles themselves do not provide detailed instructions for the implementation of provenance standards and documentation, the ISO 23494 series is intended for the provenance of data and biological samples and will be built on the World Wide Web Consortium's (W3C) PROV model,^35^ a generic provenance information standard that defines a general model, corresponding serializations† and other supporting specifications to enable the interoperable exchange of provenance information between data environments. W3C PROV serves as a framework that is adaptable and extensible to fit the needs of diverse domains. The W3C PROV standard has already been adopted in life science research areas,^36^ for example, for computational workflows,^37^ pharmacologic pipelines,^38^ neuroscience,^39^, ^40^ microscopy experiments,^41^ medical sciences,^42^ and health implementation care‡ in HL7 FHIR.^43^ Unfortunately, these implementations occurred without coordination and the resulting solutions are often incompatible, incomplete, expressed at different levels of granularity, and do not use a consistent approach for creating a continuous chain of provenance from the “source” to the resulting data. Instead of redefining the W3C PROV concepts, we have identified gaps that need to be filled to develop a distributed, fully technically and semantically interoperable provenance information standard that covers uninterrupted documentation of the whole life cycle of a dataset back to its “source”. The “source” can include a complex, multi‐institutional environment and can be both the source specimen and data, but also a link to a specific biological entity, or environmental specimen collected at a given time and location (*connectivity* requirement^44^).

**FIGURE 1:**
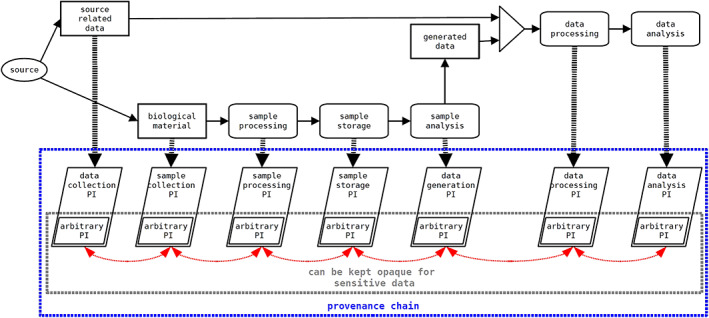
An overview of the provenance chain. A sample obtained from a donor (or other sources) is created and an initial set of provenance information (PI) is generated. As that sample moves through time and space, is processed and/or analyzed, additional provenance data are appended to the provenance chain for each new item. The chain can be extended as a complete unit of later stages of provenance or use unique identifiers to refer to early stages of provenance data. The figure cited from.^33^

The main goals of the provenance information standard are as follows:

- To support improved traceability and reproducibility of life‐sciences research, to provide a voluntary provenance framework enabling concordance of governments, businesses, academia, and the international community.
- To enable decision‐making about the fitness‐for‐purpose of particular data and specimens, by collecting and linking provenance information from the whole life cycle of the object (from specimen collection and processing, through data generation and analysis) as depicted in Figure 1.
- To achieve harmonization of documentation of specimens that is compliant with international conventions, recognized ethical practices, and legal requirements such as the Nagoya Protocol^45^ and the Declaration of Taipei.^46^

The standard will enhance the trustworthiness of provenance information by including requirements and guidelines on its integrity, authenticity, and non‐repudiation,^47^ to prevent the production and/or use of unreliable, flawed, or fabricated data (the potential harms of which have become evident also during the COVID‐19 pandemic),^2^, ^14^ as well as accidental or malicious modification of data. Since provenance information may also include sensitive or personal data (related, eg, to the health condition of an individual), the standard aims to enable sensitive information to be concealed and disclosed only under strictly controlled conditions, while preserving its core properties of integrity, authenticity, and non‐repudiation. Additional advanced application scenarios include tracking of provenance information to (i) track research error propagation, (ii) identify people affected by incidental research findings, (iii) check compliance with applicable regulations, or (iv) support the production of reference material by maintaining full documentation of provenance (complementing work of ISO/TC 334^48^). For research concerned with highly regulated fields in life sciences, such as the development of medical products or drugs, the standardized provenance model will also contribute to a level of accountability and auditability of research organizations.

The proposed standard is designed to cover the majority of the organizations involved in life‐sciences research, both academic and industrial, government labs, and research centers. Included organizations are university and industrial research laboratories, hospitals, biobanks and biorepositories, culture collections, research centers, and private companies (eg, pharmaceutical companies or lab reagent suppliers). The broader audience includes not only research data producers, but also those publishing, cataloging, archiving, or reusing research data.^49^ The standard can also be adopted by manufacturers and vendors of laboratory instruments—for example, automation devices, microscopes, sequencers, spectrometers—to enable automated standard‐compliant generation of provenance information. Automated generation of provenance information will minimize human errors and the burden put on workers, both in terms of effort and training. Provenance information generated automatically by devices should be interoperable to enable automated integration and quality control as well as validity checks demonstrating standard‐compliant provenance. The standard is intended to cover a wide range of research and applications in life sciences and for that reason, a modular structure has been used to enable extensibility to evolving requirements, processes, or technologies.

The current draft proposal ISO/DTS 23494‐1 is the first part of a planned series of six parts, with the intent that each will become a distinct ISO standard:

1. *Design concepts and general requirements* provide general requirements on provenance information management, thus enabling interconnections between the various components of provenance information in distributed environments. It also specifies requirements applicable to entities responsible for generating the provenance information.
2. *The Common Provenance Model* builds on the W3C PROV model, defining representations of elements common to all stages of research, such as the interlinking of distributed components of provenance information, the identification of physical and digital objects, provenance information patterns for common scenarios, such as missing provenance components in the chain, the compound processes, versioning of provenance information, or documentation of accountabilities. The model will also define mechanisms to embed or reference entire records of provenance information.
3. *Provenance of Biological Material* defines the requirements and scope of the provenance information documenting biological material or specimen acquisition, handling, and processing and builds on the Common Provenance Model. This includes, but is not limited to, data on collection and collection procedure, transport conditions, and documentation of the legal and ethical basis (eg, consent, terms of access, and benefit sharing) of the collection. It will also provide mechanisms to reference Standard Operating Procedures and compliance with or deviations from them. Referencing the widely accepted de‐facto reporting standard for biological specimen quality SPREC^50^ will also be enabled. Actual techniques or practices for handling biological material are not specified in the standard, in favor of technical specifications enabling consistent interoperable and machine‐actionable documentation of handling biological material. With the provenance information provided, however, the standard facilitates the verification of compliance with other pre‐analytical ISO standards covering biobanking, analytical and processing methods, and generation of reference material and related fields (ISO 20387:2018, ISO 20184 series, ISO 20166 series, and ISO 20186 series).
4. *Provenance of Data Generation* defines the provenance of data generated from the analysis or observation of biological material, for example, sequencing, microscopy, spectrometry, and so on. Provenance information specific for diverse analytical or observational data generation methods will be embedded in a way meeting the requirements of a particular domain, but is well compliant with the provenance model standard allowing seamless integration in a complete provenance chain. This will be supported by the definition of standardized links from provenance to domain‐specific information documenting the applied data generation method. As the syntax and semantics of the domain‐specific information may be in the scope of another standard, the standardized links will provide information about the conformance of the domain‐specific information to a particular standard.
5. *Provenance of Data Processing* defines the provenance of computational aspects of life sciences research such as the execution of computational workflows, for which we plan to leverage existing standards such as CWLProv^37^ and RO‐Crate,^51^ which is being complemented by a specialized profile to capture the provenance of workflow runs.§
6. *Security Extensions* define optional extensions supporting authenticity, integrity, and non‐repudiation of provenance information, and hence its trustworthiness and reliability. Demonstration of these properties will also be supported for sensitive elements of provenance information.

The ISO standards development process responds to a market need and is based on globally relevant expertise. The product is a voluntary consensus standard developed through a multi‐stakeholder process. ISO/DTS 23494‐1 and ISO/PWI TS 23494‐2 have a proven market need and have passed through the preliminary stages of the ISO voting process—as a result, they are part of the ISO Work Programme. ISO/DTS 23494‐1 *Provenance information model for biological material and data—Part 1: Design concepts and general requirements* is published. Part 2 of this series, *Biotechnology*—*Provenance information model for biological material and data*—*Part 2: Common provenance model*, has been accepted by ISO/TC 276/WG 5 as preliminary work item ISO/PWI TS 23494‐2. Part 3 of the series, *Biotechnology*—*Provenance information model for biological material and data*—*Part 3: Provenance of biological material*, will be proposed to become a Preliminary Work Item in 2023. The future documents in this series are in the planning stages but have not yet been submitted to ISO/TC 276/WG 5. The standards development process builds on existing standards for the collection and processing of specimens, analytical techniques, and data generation and analysis, as well as use‐cases from the biomedical domain. BBMRI‐ERIC, which is also active in developing international standards for biobanking, has drafted use‐cases for biological material provenance. Collaborations and ISO liaisons with professional societies like the European, Middle Eastern and African Society for Biobanking (ESBB) and the International Society for Biological and Environmental Repositories (ISBER) have also contributed to the development of specimen provenance use cases. In addition, use cases on data generation and processing can come from subject matter experts and the scientific community including the European EOSC‐Life project,¶ Open Microscopy Environment, OME,** genetic data compression (ISO/IEC JTC1/SC 29/WG 08 MPEG‐G),^52^ clinical trials and decision support systems and other life sciences domains such as biodiversity, marine biology, and systems biology.

### 2.1 Industrial vs community‐based standards

Alternatives to the ISO standards process†† exist—some community‐based efforts have developed widely adopted specifications that have become de facto global standards.‡‡ The success of these examples lies, at least in part, in the pairing of a specification with an accessible implementation that validates the utility of the specification and allows a broad community to explore integration into applications that extend far beyond the initial target.^56^ We believe that community‐led and ISO‐based approaches for developing and delivering standards can complement each other and that a combination of parallel efforts for developing a provenance chain standard might ultimately be the most productive approach. As the provenance information model development is grounded in the EOSC‐Life project, collaboration with these communities is already established. Industrial collaboration is established by grounding the standardization effort in the ISO, where industry experts drive all aspects of a standard development process through their involvement in the ISO Technical Committees. The presented ISO standard development is thus considered a standardized instance of a publicly available provenance model^33^ developed in parallel under the auspices of the EOSC‐Life project.^57^

Another challenge is the continuous dissemination and periodic revision of the standard once published. Though ISO standards are not “open access,” they can be purchased for a moderate fee§§ or accessed through institutional libraries, and, barring any patent restrictions, can be freely implemented, for instance, in Open Source software. ISO standards can also include Open Source reference implementations as specific normative or informative parts of the standards. ISO standards can be implemented independently or based on such source code, in compliance with the reasonable and non‐discriminatory (RAND) licensing terms imposed by the ISO requirements. Such licensing terms, like for instance the one applied to all ISO/IEC/SC29 (MPEG) standards that are free from any charge for scientific and non‐profit research purposes, may or may not include licensing fees.

### 2.2 Open issues

The Common Provenance Model can be seen as a current state‐of‐the‐art provenance model for distributed provenance, which is the most advanced provenance model that aims to provide a foundation for distributed provenance chains.^33^ The development of the CPM was piloted using a distributed research pipeline covering biological material acquisition and storage, sample processing, data generation, and data processing. The prototype implementation of provenance generation was provided for the computational steps of the research pipeline.

However, the model should be rigorously validated in different domains, including multiple scientific communities and industries, to verify its applicability in diverse domains in life sciences. The model is currently being applied in the BY‐COVID project,¶¶ which aims to develop a platform to integrate sources related to viral infections (clinical data, biological material, and research results). As part of this activity, the model will be integrated with RO‐Crate^51^ and applied to various use cases, including machine learning computational workflows and federated analysis.

We would like to invite experts from biotechnology and biomedical fields to further contribute to the standard, in particular to the provenance of biological specimens, the data generation, and data‐processing modules. Help is needed to develop applications of the general modules and the development of specific use cases, as well as direct contributions to the text of the standard itself. Contributions are possible through a liaison organization, a national ISO body, or by engaging with BBMRI‐ERIC.

## CONFLICT OF INTEREST STATEMENT

The authors report that they have no conflicts of interest.

## Endnotes

* Principle R1.2: (Meta)data are associated with detailed provenance.

† As defined in ISO 21597‐1:2020: encoding of an ontology or dataset into a format that can be stored, typically in a file.

‡
https://www.hl7.org/fhir/provenance.html.

§
https://www.researchobject.org/workflow-run-crate/.

¶
https://www.eosc-life.eu/.

**
https://www.openmicroscopy.org/.

††
https://www.iso.org/developing-standards.html.

‡‡ E.g., for on‐line cryptography (RSA public keys^53^), scientific workflows (Common Workflow Language^54^) and bioimaging data formats (OME‐TIFF^55^).

§§ In some cases ISO standards can be obtained without any fee, for example, https://www.iso.org/covid19.

¶¶
https://by-covid.org/.
